# Impact of the Pre-Operative Standardized Nutritional Protocol in Infants with Congenital Heart Disease (CHD)

**DOI:** 10.3390/jcdd12050166

**Published:** 2025-04-23

**Authors:** Patrick Zacharias, Jenna Blinci, Ruthie Shenoy, Jesse Lee, Yogen Singh

**Affiliations:** 1 Department of Pediatrics, Loma Linda University, Loma Linda, CA 92354, USA; pzacharias23@gmail.com (P.Z.); jmabrassart85@gmail.com (J.B.); ruthieshenoy@gmail.com (R.S.); 2 Department of Pediatrics, Division of Cardiology, Loma Linda University, Loma Linda, CA 92354, USA; jwlee@llu.edu; 3 Department of Pediatrics, Division of Neonatology, University of California Davis, Sacramento, CA 95817, USA

**Keywords:** congenital heart defects, nutrition, protocol, necrotizing colitis (NEC), growth

## Abstract

Neonates with congenital heart disease (CHD) are at increased risk of growth failure and necrotizing enterocolitis (NEC), making nutritional management crucial for their outcomes. This study aimed to evaluate the impact of a standardized feeding protocol on growth and NEC incidence in CHD infants. A retrospective study was conducted at a tertiary care center, including neonates diagnosed with CHDs from January 2020 to March 2023. Patients were divided into two groups: those receiving the standardized feeding protocol (protocol group, *n* = 12) and those who did not (non-protocol group, *n* = 39). Key metrics such as growth velocity at discharge, anthropometric z score changes at discharge since birth, days to full enteral feeds, NEC incidence, and length of stay were analyzed. Statistical comparisons were made using two-tailed Mann-Whitney test and chi-squared tests. The NEC incidence was 10% in the non-protocol group and 0% in the protocol group (*p* = 0.25), with no significant difference. All anthropometric growth markers at the time of discharge differed between the groups, with the protocol group demonstrating favorable outcomes across all measured variables; however, these differences did not reach statistical significance. The time to reach full enteral feeds was shorter (8.5 days vs. 11 days; *p* = 0.22), and length of stay was shorter in the protocol group (17 days vs. 23 days; *p* = 0.14), although neither was statistically significant. Although the protocol group showed trends towards reduced NEC and improved growth, this was not statistically significant, which could have been because of the small sample size. Our findings suggest that a standardized feeding protocol may reduce the time to full enteral feeds and hospital stay, but further large-scale studies are needed to confirm these results.

## 1. Introduction

Nutrition is of paramount importance for neonates with congenital heart disease (CHD). The intricacies of nutrition delivery in this population have been widely studied and have led to the development of protocolized nutritional regimens that have been adopted by neonatal intensive care units (NICUs) in recent years. Neonates affected by CHD during the pre-operative period were found to have an increase in total energy expenditure—upwards of 30% compared to healthy controls [1,2,3]. Most were found to have protein deficits and overall “growth failure”, defined as weight-for-age decreasing across two major percentile channels from a previously established growth pattern or weight-for-length < 80% of ideal body weight, demonstrating the importance of maximizing nutrition particularly in this group [4,5].

Studies have mainly focused on infants with hypoplastic left heart syndrome (HLHS), and they have demonstrated that infants who were given adequate pre-op feedings had shorter mechanical ventilation time, less fluid overload, and earlier post-op feeding tolerance [4,6,7]. The hesitancy in providing enteral feeding in the HLHS cohort has been due to concerns over necrotizing enterocolitis (NEC) risk. The risk of NEC in infants with CHD has been evaluated in multiple studies, which have a found higher incidence of NEC in infants with HLHS, total anomalous pulmonary venous return (TAPVR), truncus arteriosus (TA), unbalanced atrio-ventricular septal canal defects (AVSDs), Tetralogy of Fallot (ToF), patent ductus arteriosus (PDA), and atrial septal defect (secundum type) (ASD). However, more recent studies suggest that a delay in feeding itself may increase the risk and severity of NEC [1,8,9]. This has been explained based on the fact that a lack of enteral feeding leads to the rapid atrophy of the intestinal mucosa, with loss of the critical barrier function [1,8]. Even minimal enteral nutrition (MEN), typically defined as 10 to 20 mL/kg/d, was found to aid in intestinal mucosa development and the maturation of its associated immune system, and has been associated with a decreased incidence of NEC in this population [1,6,7]. The balance of nutritional delivery in neonates remains an important topic of research. Further investigation into nutritional delivery via protocolized feeding regimens, the benefits of those regimens in supporting growth and minimizing unwanted complications, and ultimately decreasing the risk of the potentially lethal complication of NEC in this already high-risk population is needed.

In our institution, the feeding protocol was developed via a collaborative approach between neonatologists, pediatric cardiologists, and dieticians. It focused on establishing feeding in infants with CHD, and was developed with an aim to optimize nutrition and decrease the incidence of NEC. The aim of our study was to evaluate the impact of the implementation of the special feeding protocol for the infants with CHD on the reduction in NEC, optimize overall growth, and study the impact on other outcomes, such as length of stay and days to full feeds.

## 2. Materials and Methods

A retrospective observational study involved chart analysis for all the patients diagnosed with congenital heart disease at our institution from January 2020 to March 2023. The following ICD-10 codes were utilized in the search: 135.0, Q20.0, Q20.1, Q20.3, Q20.4, Q21.0, Q21.12, Q21.3, Q22.4, Q23.0, Q23.4, Q24.9, Q25.0, Q25.1, Q25.5, Q26.2, and Q26.3. These codes allowed us to capture infants born with HLHS, Tetralogy of Fallot (ToF) with or without pulmonary atresia, atrio-ventricular septal defect (AVSD), double outlet right ventricles (DORV), transposition of the great arteries (TGA), large patent ductus arteriosus (PDA), ventricular septal defects (VSD), coarctation of the aorta, or severe valvular disease. The exclusion criteria were as follows: infants with congenital heart defects not requiring surgical correction (ex: insignificant VSD or ASD, PFO, etc.) and those with a NICU stay of less than 7 days.

**Figure 1:**
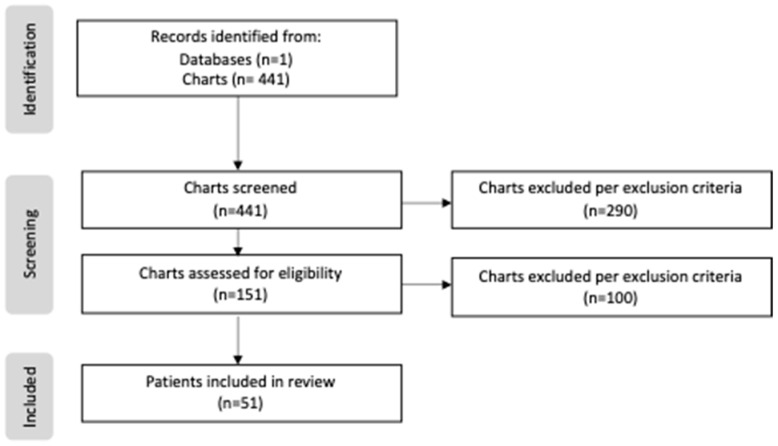
PRISMA flow diagram of study.

Patients born prior to the creation of our center’s feeding protocol were categorized as “non-protocol”, as well as those patients with which no protocol was utilized despite the institutionalization of the protocol. Those patients who successfully started on the created protocol were categorized as “protocol”. A total of 441 patients were screened and reviewed, including 274 patients in the non-protocol group and 167 patients in the protocol group. During the screening process, only 151 patients of the 441 met the study criteria, as the excluded cases either had lower-risk CHD that did not require surgical repair, or were transferred out of the NICU before day 7 of life. Of the 151 eligible cases, an additional 100 patients were excluded due to limited data or a lack of enteral intake. Following these exclusions, the total study sample included 39 patients in the non-protocol group and 12 patients in the protocol group (Figure 1).

**Figure 2:**
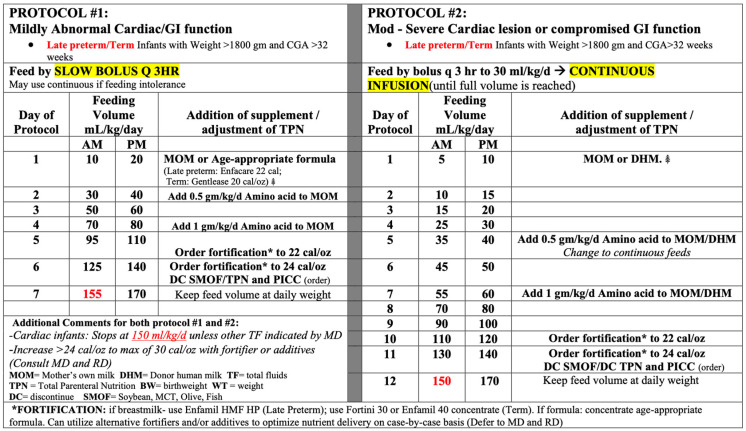
Summary of the feeding protocols.

The institutional cardiac feeding protocols that were utilized are shown in Figure 1, with the full details included in the Supplementary Material. The protocols were distinguished by the degree of abnormal cardiac function, which determined the rate of advancement. Protocol #1 focused on mild abnormal cardiac function, resulting in quicker advancement, achieving full feeds by day 7 of life (assuming feeds were tolerated). Protocol #2 focused on moderate to severe abnormal cardiac function, resulting in slower advancement, with the initiation of feeds less than trophic (20 mL/kg/d), achieving full feeds by day 12 of life (assuming feeds were tolerated). Each protocol was established for late preterm or term infants who were >1800 g at birth. If the patient was <1800 g or <33 weeks, the established institutional preterm feeding protocols were utilized based on birthweight for mild abnormal cardiac function. In the case of moderate to severe abnormal cardiac function, the protocol for lower than birthweight was utilized (i.e., a 1525 g patient at birth who would typically be started on the 1501–2000 g protocol was started on 1251–1500 g protocol due to the moderate to severe cardiac function (Figure 2)).

The following metrics were collected and analyzed: demographics, growth velocity at discharge, anthropometric z score measurement changes since birth, incidence of NEC, days to full feeds, and length of stay. Growth velocity (g/kg/d) was calculated using the exponential method = 1000 × ln (discharge weight − weight at regain of birthweight)/(day of life at discharge − day of life at regain), where ‘ln’ is the natural logarithm and weight is expressed in grams. Anthropometric z score measurements at birth and at discharge for weight, length, and head circumference were obtained and compared to the “change from birth” to assess the overall growth. In the case of unavailable anthropometrics, measurements closest to the desired day were utilized in the assessment. For example, in the case of missing weights, the weight measurement +1 or −1 from the desired day was used. For both length and head circumference measurements (which are obtained weekly per unit standard), the last measurement closest to the desired day was used. “Full feeds” were defined as reaching an intake of 150 mL/kg/d enterally. The length of stay was assessed from the day of birth (identified as DOL 1) to the date of discharge or transfer to Cardiothoracic ICU (CTICU) for surgical intervention.

Statistical analysis was completed using the two-tailed Mann–Whitney test, given our small numbers in each study group for quantitative variables and using the chi-squared test for categorical variables such as NEC incidence. The study was approved by the Institutional Board Review of Loma Linda University of Health.

## 3. Results

### 3.1. Demographics

The study demographics ultimately included 39 patients in the non-protocol group and 12 patients in the protocol group. The percentages of patients that were born preterm (<37 weeks) were, respectively, 43.6% for non-protocol and 41.7% for protocol, with similar average gestational ages of 36.2 weeks (non-protocol cohort) compared to 36.5 weeks (protocol cohort). The average birth weight was also similar between groups with 2740 g for non-protocol and 2770 g for protocol groups. The incidence of HLHS and critical stenosis or coarctation in each group was 17.9% and 16.7%, for the non-protocol vs. protocol group, respectively (Table 1a). There were no significant differences in ethnicities between the two study groups.

### 3.2. Incidence of NEC

The incidence of NEC was 10% (*n* = 4) for the non-protocol group compared with 0% in the protocol group (*p*-value = 0.25) (see Table 1a). The characteristics of the patients are shown in Table 2.

### 3.3. Nutrition and Growth

Growth velocity at discharge was calculated with a mean of 7.4 g/kg/d for the non-protocol and 9.3 g/kg/d for the protocol groups (*p* value = 0.75). Of note, five patients were excluded in the non-protocol group and three in the protocol group, given that the birthweight was not regained and the growth velocity was calculated once the birthweight has been regained, rather than from birth. Anthropometric z score measurements at birth and at discharge for weight-for-age, length-for-age, and head circumference-for-age were compared between groups, with *p*-values of 0.52, 0.92 and 0.94 for weight, length, and head circumference change, respectively.

Day of life (DOL) when birth weight was regained was compared between the groups, resulting in an average of 8.0 days for the non-protocol group vs. 7.5 days for the protocol group (*p*-value = 0.54).

### 3.4. Achievement of Full Enteral Feeds

The day of life (DOL) when patients reached full feeds was calculated to be an average of 11 days for the non-protocol group and 8.5 days for the protocol group (*p*-value = 0.22). Notably, eight non-protocol patients and two protocol patients were omitted from these calculations due to not reaching full feeding prior to discharge/transfer to the CTICU. See Table 3 for a summary of the collected data.

### 3.5. Length of NICU Stay

The length of stay (LOS) between each group was also calculated, with an average of 23 days and 17 days for non-protocol and protocol groups, respectively, (*p*-value = 0.14).

## 4. Discussion

In the context of neonates born with congenital heart defects (CHDs), to feed or not to feed is a hotly debated topic, which lacks a consensus institutionally, nationally, and internationally. The multidisciplinary team, composed of neonatologists, cardiologists, and dieticians, recognize the significantly increased prevalence of NEC, which is widely published within this unique population. Many retrospective studies have reported a 3–5% incidence of NEC in infants with CHDs, with that incidence increasing to 6.1–9% in HLHS cases, with single ventricle defects making up 40% of the cohort per McElhinney [3,8]. Siano et al. states that 48% of NEC in CHD develops pre-operatively, which contributes to the hesitancy to feed this population [4,6,8]. However, there is a lack of convincing evidence that the increased NEC risk is correlated with pre-operative feedings, rather it is suggested that it correlates with the multifactorial nature of the pathophysiology of NEC [4,8]. Carlo et al. demonstrated an impaired mesenteric blood flow in 47% of infants with CHD who developed NEC compared to 15% in controls (OR 5.04, 95% CI 1.02–23.82, *p* < 0.05) [8]. In a comparative study by Natarajan et al., lower gestational age significantly increased NEC (23% vs. 1.7%) [8]. The care of infants with CHDs is a balance of risks and benefits. While previous studies within the CHD cohort minimize enteral delivery due to its intrinsic risk of NEC, more recent studies shift this practice, highlighting the benefits that presurgical enteral feedings can provide.

Current research continues to suggest that the benefits of feeding greatly outweigh the concerns. One study on a HLHS patient that received preoperative feeds showed that they had a shorter duration of postoperative mechanical ventilation, less fluid overload, and earlier postoperative feeding tolerance [7,10]. Standardized feeding protocols have demonstrated benefits, including reduced necrotizing enterocolitis (NEC) risks and improved clinical outcomes [1,6,11]. Gephart et al. indicated studies showing that early enteral nutrition leads to improved nutritional outcomes, decreased mechanical ventilation duration, decreased infection rates, improved wound healing, decreased length of stay, and reduced mortality [6]. Jasani et al.’s meta-analysis (18,160 infants, <2500 g or less than 1500 g) conferred a 78% reduction in NEC when a unit adopted a standardized feeding protocol (*p* > 0.00001) [12]. While neonatal intensive care units (NICUs) are familiar with feeding protocols, they may be less common in pediatric cardiac ICUs [11,13]. Although some protocols have reported a decreased incidence of NEC during the NICU stay, currently there is a lack of suggested protocols for the pre-operative infant, especially for use in pediatric cardiac intensive care units.

Our institute established feeding protocols for the pre-operative infant to optimize nutritional delivery for better growth and better outcomes (decreasing length of stay, days to full feeds, etc.). Compared to non-protocol patients, infants fed with the standardized protocol showed a lower incidence of NEC (10% vs. 0%, *p*-value = 0.25), as well as a trend toward a higher growth velocity at discharge (9.3 g/kg/d vs. 7.4 g/kg/d (*p* value = 0.75). Although these outcomes are statistically non-significant, there is a strong trend toward a reduction in NEC and an improvement in all anthropometric measurements. It is important to note that the non-protocol sample size (*n* = 12) was limited and likely affected the overall statistical significance. Jasani et al.’s meta-analysis, which did have a large sample size (18,160 infants, <2500 g or less than 1500 g), conferred a 78% reduction in NEC when a unit adopted a standardized feeding protocol (*p* < 0.00001) [12].

The use of a protocol did also suggest a trend towards reduction in the days to full feeds when compared to the non-protocol group (8.5 days vs. 11 days, *p*-value = 0.22). Another study by Boston Children’s hospital with higher power (*n* = 80 in both cohorts) reported that the use of a standardized feeding regimen significantly decreased the median time to reach energy goals from 4 days to 1 day (*p* < 0.0001), and an increased proportion of patients reaching this goal (99% vs. 61%, *p* = 0.01) [14].

Interestingly, there was an associated reduction in the length of hospital stay in the protocol group compared to the non-protocol group (17 days vs. 23 days, *p*-value = 0.14). This lack of statistical significance is likely related to the small sample size in the protocol group. Other studies have reported a reduction in hospital stay with the use of standardized feeding protocols [1,6,14].

Our study demonstrates that it is feasible to implement a standardized feeding protocol for infants with CHDs. With a multidisciplinary collaborative team approach and insights on the benefits of early feeding, a carefully implemented standardized feeding protocol may help these infants have improved growth, a decreased hospital stay, and in fact a decreased risk of NEC. The adoption of standardized protocols may also help to address institutional variability in feeding practices, which remains a barrier to widespread adoption in pediatric cardiac care.

We acknowledge the limitations of the study. (1) The sample size was very small, especially in the protocol group, to evaluate a statistically significance between the two groups, and (2) not all eligible infants followed the newly implemented feeding protocol—this was utilized in 57% of eligible cases after its development and implementation on the unit. While no objective data of why protocols were underutilized could be obtained through our chart review, we hypothesize that provider comfort/familiarity and disagreement among cardiologists and neonatologists may play a role in underutilization. Given the varying utilization of the protocols, it is important to note that there was the potential for selection bias in our study. This may have affected the study results and provided an important glimpse into the reality of the hesitancy to start implementing protocols in this population. Despite these limitations, our study demonstrated that standardized feeding protocols have the potential to decrease NEC and other comorbidities, optimize growth, and decrease hospital stay duration.

## 5. Conclusions

Our study suggests that there was an improvement in days to establishing full feeds in the infants in the protocol group compared to those in the non-protocol group, as well as a reduction in the hospital stay duration, although statistical significance was not reached. There was a trend towards reduction in the NEC and improved growth velocity at discharge in the protocol group. This could be because of the small protocol sample size affecting the statistical significance. A large prospective study to evaluate the impact of standardized feeding protocols developed for infants with congenital heart defects is urgently warranted. With a larger sample size, the correlations between feeding, risk of NEC, and growth can be reliably established.

It is well known among neonatologists, cardiologists, and dieticians that this population has higher energy expenditure needs that support the need to feed, yet also has a higher risk of NEC incidence that at times contradicts feeding. Developing and adopting feeding protocols may help to achieve this balance safely.

## Figures and Tables

**Table 1:** (**a**) Study demographics. (**b**) Cardiac lesions and presence of comorbidities.

(**a**)					
			**Non-protocol**		**Protocol**
Sample Size (*n* =)			39		12
%Preterm			43.6%		41.7%
Average GA (weeks)			36.2		36.5
Average BW (g)			2740		2770
HLHS, Critical Stenosis, Coarctation			*n* = 7 (17.9%)		*n* = 2 (16.7%)
Non-cardiac congenital defects			43.6%		16.7%
NEC incidence			10%		0%
(**b**)					
**Non-Protocol**			**Protocol**		
**Patient #**	**CHD**	**Comorbidities**	**Patient #**	**CHD**	**Comorbidities**
2	TOF + PA	DiGeorge syndrome (22q11 deletion), moderate right hydronephrosis, vesicoureteric reflux	706	TOF, subvalvar stenosis, mod valvar stenosis, ASD, PDA	None
30	Complete AV canal	Trisomy 21, duodenum inversum without malrotation	707	CoA with aortic arch, PDA, VSD, ASD	None
37	hsPDA	None	709	TOF with PS, VSD, PDA, PFO	None
47	TOF with PA with MAPCAs	Butterfly verterbra (T6)	712	Small ASDs, VSDs	None
51	D-TGA, large VSD, CoA	None	714	TOF, DORV, VSD, PFO, PV stenosis	None
58	TOF	None	738	TOF	None
67	Moderate VSD	Congenital hypothyroidism, extreme prematurity (26 weeks)	740	CoA	Turner Syndrome, horseshoe kidney
80	TOF	None	747	TOF with severe PS	None
85	Dextrocardia, Complete AV defect unbalanced, subpulmonary stenosis, TAPVR	None	749	DORV with subaortic VSD	None
91	PS	None	758	DORV with TGA, large VSD, large ASD	None
110	CoA, large PDA	None	759	DORV with TGA, mod PV stenosis	None
130	L-TGA, PS	None	800	Large VSD	Trisomy 21, right hydronephrosis, BPD
133	DORV, large VSD/ASD	None			
136	Shone’s: hypoplastic arch, VSD, ASD	None			
146	TOF with mild PS	Monosomy 7 mosaicism, global development delay, encephalomalacia			
151	TOF with severe PS	None			
164	TOF without outflow obstruction	VACTERL, microtia of left ear, hearing loss in both ears, congenital nasal pyriform aperture stenosis, lobar holoprosencephaly, absent septum pellucidum, rib deformity, horseshoe kidney, pulmonary hypoplasia			
168	Complete AV canal	Trisomy 21			
169	TOF, DORV, subaortic VSD, cortriatrum, mod RVOT	VACTERL, Esophageal atresia, spinal anomaly			
202	Large VSD, bicuspid AV, hypoplastic arch	None			
210	TOF with mod outflow obstruction	None			
211	HLHS	Heterotaxy with visceral situs inversus			
216	TOF	DiGeorge, inguinal hernia, immunodeficiency, t cell deficiency, tracheobroncomalacia			
228	AV Canal defect	Trisomy 21, aphakia, suspect glaucoma			
233	DORV, moderate pulm obstruction, right aortic arch	None			
244	Large VSD, moderate ASD	VACTERL, penile torsion, retractile testes, imperforate anus, sacral dimple			
258	TOF	None			
261	TOF	Trisomy 21, umbilical hernia			
265	Severe PV dysplasia/stenosis	None			
266	Severe CoA, mod arch hypoplasia, bicuspid AV, VSDs	Intestinal malrotation, polysplenia			
640	CoA, PFO, LSVC to CS, VSD, bicuspid AV	Pierre robin sequence, turner syndrome (Webbed neck), micrognathia, stenosis of external auditory canals			
643	CoA, tiny VSDs	Intestinal malrotation			
645	ASD, TAPVR	None			
668	DORV, VSD, PS, ASD	None			
678	Large ASD, PV stenosis	None			
682	Large VSD, bicuspid AV	Trisomy 18, cerebellar hypoplasia			
692	VSD, hsPDA	None			
697	hsPDA	None			
730	hsPDA	None			

NEC—necrotizing enterocolitis, HLHS—hypoplastic left heart syndrome, GA—gestational age, BW—birthweight in grams, CHD—congenital heart defect, AV—atrioventricular, PDA—patent ductus arteriosis, TOF—Tetralogy of Fallot, MAPCAs—Major Aortopulmonary Collateral Arteries, TAPVR—Total Anomalous Pulmonary Venous Return, VSD—ventricular septal defect, ASD—atrial septal defect, PFO—patent foramen ovule, PA—pulmonary atresia, PV—pulmonary valve, PS—pulmonary stenosis, CoA—coarctation, DORV—double outlet right ventricle, RVOT—right ventricle outflow tract.

**Table 2:** NEC patient characteristics.

Patient #	GA	BW	CHD	Congenital Anomalies
1	23w6d	650 g	hsPDA	None
2	37w1d	3345 g	DORV, large VSD/ASD	None
3	38w6d	2630 g	TOF with mild PS	Monosomy 7 mosaicism, global developmental delay, encephalomalacia
4	30w0d	1270 g	TOF without outflow obstruction	VACTERL, microtia of left ear, hearing loss in both ears, congenital nasal pyriform aperature stenosis, lobar holoprosencephaly, absent septum pellucidum, rib deformity, horseshoe kidney, pulmonary hypoplasia

**Table 3:** Summary of collected data.

		Non-Protocol				Protocol			
	Sample Size	Median	25%	75%	Sample Size	Median	25%	75%	*p* Value
GV at discharge	34 *	7.3	2.9	12.55	9 *	9.3	4.7	10.9	0.75
WAZ change at discharge	39	−0.75	−1.16	−0.36	12	−0.62	−1.15	−0.05	0.52
LAZ change at discharge	39	−0.83	−1.69	−0.05	12	−0.51	−1.22	−0.17	0.92
HAZ change at discharge	39	−0.76	−1.64	−0.25	12	−0.61	−1.1	−0.04	0.93
DOL when regained Birthweight	34 *	8.0	7.0	11.0	9 *	7.5	5.25	10.5	0.54
Length of stay	39	23	14	47	12	17	11.5	23	0.13
Day of life when full feed reached	31 ^+^	11	7	23	10	8.5	7.5	11.5	0.21

GV stands for growth velocity, WAZ for weight-for-age z score, LAZ for length-for-age z score, HAZ for head circumference-for-age z score; * five patients excluded from non-protocol and three from protocol due to birthweight not being regained; and ^+^ eight patients excluded from non-protocol and two from protocol due to not reaching full feeds.

## Data Availability

The original contributions presented in this study are included in the article/Supplementary Materials. Further inquiries can be directed to the corresponding author.

## Supplementary Materials

The following supporting information can be downloaded at: https://www.mdpi.com/article/10.3390/jcdd12050166/s1.
