# Supplementary material for: Impact of the Pre-Operative Standardized Nutritional Protocol in Infants with Congenital Heart Disease (CHD)
Source: J Cardiovasc Dev Dis. 2025 Apr 23;12(5):166. doi: 10.3390/jcdd12050166 (PMC12112651; doi:10.3390/jcdd12050166)
Supplement: Supplementary file 1 [file jcdd-12-00166-s001.zip › jcdd-3409571-supplementary.pdf]

# Enteral Feeding Guidelines for Neonates with Congenital Heart Lesions or Post-surgical
## July 2022

**AIM**: Using standardized approach for feeding initiation and advancement in GI surgical patients and cardiac neonates to achieve:

- improved tolerance
- better growth, achieved by the following nutritional aims to achieve weight gain of >15 g/kg/day
    - Term: 100-120 cal/kg/day and 2-3.5 g/kg/day
    - Preterm: 120-150+ cal/kg/day and 4.5+ g/kg/day
- decreased time on the ventilator, incidence of NEC, sepsis and shorten length of stay

**GOAL: initiate feeding asap as there is evidence of bowel function, when** infant:

- is hemodynamically stable and has stooled
- Postsurgical patient: sump output is no longer dark bilious (If output is mostly clear, dc sump suction for 24h. If no emesis consider starting trophic feeds) Feeding start is a joint decision for surgical patients(peds surgery and neonatology discussion)

**Contraindications** to feedings include:

- GI perfusion/oxygenation suboptimal: Hemodynamically significant cardiac disease with high pressor support, poor perfusion, lactic acidosis, polycythemia, and refractory hypoxemia,

**Discontinue feeds*** or decrease volume/concentration/ if there is feeding intolerance Follow Feeding Intolerance Algorithm

**Feeding intolerance defined as**:

- increased ostomy/stool output,
- increased emesis/dark bilious emesis, diarrhea (liquid >10/day),
- significant abdominal distention above baseline (>10%)
- abdominal tenderness/significant bowel loops.
- abnormal vital signs

**If mild intolerance, hold feeds for 12 hours**, reassess and restart if improved.
**Xray evaluation** prior to restarting feeds if dark bilious emesis, hematochezia or concerning abdominal exam
If bowel is significantly dilated (over baseline) on KUB→place sump to suction and observe. If no significant output stop suction and consider reintroduction of feeds **in 12- 24 hours**.

*If intolerance is not serious may also consider changing feeds to **Minimal Enteral Stimulation**, aimed not to feed but to keep mucosal integrity: 0.1-0.5 ml/hr of Breast Milk by continuous drip.

# FEEDING GUIDELINE FOR THE CARDIAC/SURGICAL NEONATE

| **Feeding RISK Classifications** | **Anticipated CARDIAC/ GI FUNCTION** | **Diet** | **Feeding Schedule** | **Advancement** | **Additional Therapy** |
|---|---|---|---|---|---|
| I Normal to Mildly abnormal | Normal to Mildly Abnormal<br><br>**Use Feeding Protocol #1** | MOM/ Std Formula<br>PE24 for premie<br>E20 for term<br>GE 20 for cardiac term<br><br>DHM or Pregestimil/ Nutramigen if intolerance to age-appropriate formula | Start Bolus Q 3 Hr feedings<br><br>Consider Continuous if:<br>- Intolerance | Start 10-20cc/kg/d<br><br>Advance by 10-20/kg/d<br><br>Early protein supplementation<br><br>Fortify to 22 cal at TF 95-110<br>Fortify to 24 cal at TF 125-140 | Use SMOF lipid emulsion |
| II Abnormal | A-Moderately Abnormal<br><br>**Use Feeding Protocol #1 but may need to use #2** | MOM / DHM.<br>If not tolerated<br>↓<br>Pregestimil/Nutramigen<br>↓<br><br>If not tolerated<br>Elecare/ Puramino/ Neocate | Start bolus q 3 hr feeds to 30 ml/kg then change to Continuous feedings until full feeds<br><br>Condense feeds after tolerating full continuous feeding Starting at infusion over 90-120 minutes q 3, decreasing by 30 minutes each day as tolerated, after reaching full volume feeds<br><br>When condensing feeds consider to start nippling | Start 5-10cc/kd/d<br><br>Advance by 5-10cc/kg/d or 10mL/kg every other day<br><br>Early protein supplementation<br><br>Fortify to 22 cal at TF110-120<br><br>Fortify to 24 cal at TF 130-140 | SMOF lipid emulsion<br><br>Change to Omegaven if cholestasis (direct bili ≥ ≥5, but earlier if Short bowel Sd/high risk for liver disease)<br><br>For Short Bowel Sd Consider PPI and bacterial overgrowth management |
| | B-Severely Abnormal<br><br>**Use Feeding Protocol #2** | MOM/DHM<br>↓<br>Elecare/ Puramino/ Neocate | | | |

**<u>Examples of Classification Groups:</u>**

Class I: <u>CARDIAC</u>: PDA, VSD, ACV, TA, TGA, PS/PA
<u>SURGICAL:</u> TEF, CDH, Hirschsprung's, Anorectal anomalies, NEC in colon only, Gastroschisis with essentially normal appearing bowel, Malrotation with or without Volvulus with pink healthy bowel, NEC resection with <30% bowel resected and distal ostomy

Class II: <u>CARDIAC</u>: **A:** Single Ventricle physiology, Truncus, HLHS **B:** Class I Cardiac + perfusion issues or feeding intolerance hx
<u>SURGICAL</u>:

A. Gastroschisis with abnormal appearing bowel (dilated, thickened, ischemic, peel), Duodenal stenosis & atresias, Malrotation with volvulus with ischemic but viable bowel (no bowel resected or minimal bowel resection), NEC with extensive disease but minimal resection, jejunal or ileal atresia with expected dysmotility
B. NEC with resection >50 % small bowel remaining but bowel normal, proximal small bowel ostomy (i.e., jejunostomy), gastroschisis with severely affected small bowel, atresia or volvulus with extensive damage, high ostomy with ischemic/edematous proximal bowel with expected poor motility and/or absorption

**AUDIT: Feeding Risk Classification** (I or II) **ought to be documented in the initial note following discussion with cardiology or peds surgery.**

## PROTOCOL #1:
## Mildly Abnormal Cardiac/GI function

- Late preterm/Term Infants with Weight >1800 gm and CGA >32 weeks

**Feed by SLOW BOLUS Q 3HR**
May use continuous if feeding intolerance

| Day of Protocol | Feeding Volume mL/kg/day | | Addition of supplement / adjustment of TPN |
|---|---|---|---|
| | AM | PM | |
| 1 | 10 | 20 | **MOM or Age-appropriate formula** (Late preterm: Enfacare 22 cal; Term: Gentlease 20 cal/oz) ⸙ |
| 2 | 30 | 40 | **Add 0.5 gm/kg/d Amino acid to MOM** |
| 3 | 50 | 60 | |
| 4 | 70 | 80 | **Add 1 gm/kg/d Amino acid to MOM** |
| 5 | 95 | 110 | **Order fortification* to 22 cal/oz** |
| 6 | 125 | 140 | **Order fortification* to 24 cal/oz**<br>**DC SMOF/TPN and PICC** (order) |
| 7 | 155 | 170 | Keep feed volume at daily weight |
| | | | |

## PROTOCOL #2:
## Mod - Severe Cardiac lesion or compromised GI function

- Late preterm/Term Infants with Weight >1800 gm and CGA>32 weeks

**Feed by bolus q 3 hr to 30 ml/kg/d → CONTINUOUS INFUSION**(until full volume is reached)

| Day of Protocol | Feeding Volume mL/kg/day | | Addition of supplement / adjustment of TPN |
|---|---|---|---|
| | AM | PM | |
| 1 | 5 | 10 | **MOM or DHM.** ⸙ |
| 2 | 10 | 15 | |
| 3 | 15 | 20 | |
| 4 | 25 | 30 | |
| 5 | 35 | 40 | **Add 0.5 gm/kg/d Amino acid to MOM/DHM**<br>*Change to continuous feeds* |
| 6 | 45 | 50 | |
| 7 | 55 | 60 | **Add 1 gm/kg/d Amino acid to MOM/DHM** |
| 8 | 70 | 80 | |
| 9 | 90 | 100 | |
| 10 | 110 | 120 | **Order fortification* to 22 cal/oz** |
| 11 | 130 | 140 | **Order fortification* to 24 cal/oz**<br>**DC SMOF/DC TPN and PICC** (order) |
| 12 | 150 | 170 | Keep feed volume at daily weight |

**Additional Comments for both protocol #1 and #2:**

*-Cardiac infants: Stops at 150 ml/kg/d unless other TF indicated by MD*

*-Increase >24 cal/oz to max of 30 cal/oz with fortifier or additives (Consult MD and RD)*

**MOM**= Mother's own milk **DHM**= Donor human milk **TF**= total fluids
**TPN** = Total Parenteral Nutrition **BW**= birthweight **WT** = weight
**DC**= discontinue **SMOF**= Soybean, MCT, Olive, Fish

***FORTIFICATION:** if breastmilk- use Enfamil HMF HP (Late Preterm); use Fortini 30 or Enfamil 40 concentrate (Term). If formula: concentrate age-appropriate formula. Can utilize alternative fortifiers and/or additives to optimize nutrient delivery on case-by-case basis (Defer to MD and RD)

- **Infants with extremely severe dysfunction (Cardiac or Surgical) can be started at lower volume than DOL 1** (MES of 0.1-0.5 ml/hr continuous drip) for 1-3 days prior to starting protocol to increase time to full feeds.
- Infants with BW <1800 g and <33 weeks of GA with surgical or cardiac issues will be **started on Preterm NICU protocol (<750-2500 g)** using **actual BW** for **mildly affected** and a **protocol lower than BW** for **severely affected preterm infants.**
- Use BW for volume calculations until BW regained. Once regained → ON TPN: use calculation WT. OFF TPN: use current WT
  - If current WT is > calculation WT by 10% (not due to edema), call MD/NNP for updated calc WT. Do not decrease volume if there is WT loss.
- **At >31 weeks CGA,** start documenting **Feeding Readiness Scores** q 12 hours (if extubated) and request order for OT feeding assessment to start **cue-based nippling.** Document feeding readiness with each feeding once nippling is started.
- When **nippling well (> 80% of feeds** for 24 hr), do a **trial of nipple only** feeds, ad lib on demand (max of 4 hr between feeds).
- ⸙ May use DHM until full volume of fortified feedings has been tolerated for at least 48 hours then transition to appropriate formula over 2 to several days, according to tolerance.

- **Check Urinary Na** in patients with hyponatremia or poor growth: If urinary Na <10mEq/L or Na 10-20 mEq/L +Failure to Thrive- start supplement.
- **Consider Zn Supplement** (in presence of poor growth despite adequate caloric intake with skin rash and high ostomy output)

## MILK/FORMULA CHOICE:

- MOM (Mother's own milk) is preferred when available and tolerated.
- DHM (Donor human milk) can also be used (preferably higher risk infants)
- **Late Preterm formula:**
    - <u>Enfacare 22 cal/oz:</u> Best option for late preterm if breast milk not available. 80:20 whey:casein ratio, 20% MCT content with low osmolality (220 mOsm/kg ready to feed formulation)
    - <u>Enfamil Premature 24 (PE 24):</u> more nutritionally appropriate for small late preterm infants. 80:20 Whey:Casein ratio, 40% MCT content with moderate osmolality (300 mOsm/kg)
- **Term formula**:
    - <u>Gentlease</u>: well tolerated, low lactose, 100% partially hydrolyzed whey (60:40 whey:casein ratio), and available at 20-30 cal/oz with preference of concentrating with powder to maintain low osmolality). *Best option for term infants if no breast milk.*
    - <u>Fortini</u>: term energy-dense infant formula (30 cal/oz), 2.6 g/100cal protein delivery with low osmolarity of 360 mOsm/kg (not on formulary yet)
- **Semi elemental/Hydrolyzed formula** (⸙ Protocol #1- use this if intolerance to age-appropriate formula)
    - <u>Pregestimil</u>: containing 55% MCT which is preferred for malabsorption
    - <u>Nutramigen:</u> outpatient formulation contains LGG (probiotic)
    - <u>Alimentum</u>: contains 33% MCT content
- **Elemental powder formulas** (Elecare, Neocate, and Puramino (becoming part of formulary) may be needed for Class IIB or Class I and IIA ⸙ if intolerance to breast milk/Pregestimil

***Weaning from special formulas and from Donor HM** (if above Wgt 1800)**: ONCE GOOD TOLERANCE OF FULL/Fortified FEEDS for 2 days:**

- On Donor human milk or elemental formula (Neocate/Elecare) → transition to hydrolyzed formula (Pregestimil/Nutramigen) over 2- 4 days.
    - **Day 1**: 75% DHM/Elemental+ 25% Hydrolyzed formula
    - **Day 2**: 50% DHM/Elemental+ 50% Hydrolyzed
    - **Day 3:** 25%DHM/Elemental+ 75% Hydrolyzed
    - **Day 4:** 100% Hydrolyzed
- Once tolerating semi-elemental hydrolyzed formula → transition to age-appropriate formula days prior to discharge (over 2 to 4 days).
    - **Day 1:** 50% Hydrolyzed + 50% Age-appropriate formula
    - **Day 2:** 100% age-appropriate formula
